# Braking Parkinson’s progression: the hypothetical druggable role of striatal parvalbumin interneurons

**DOI:** 10.1038/s41531-026-01303-0

**Published:** 2026-04-08

**Authors:** Quansheng He, Xuan Wang, Xiaowen Zhang, Yousheng Shu

**Affiliations:** 1 Department of Neurology, Affiliated Hospital of Jiangsu University, Zhenjiang, Jiangsu 212001 China; 2 Institute for Translational Brain Research, State Key Laboratory of Medical Neurobiology, MOE Frontiers Center for Brain Science, MOE Innovative Center for New Drug Development of Immune Inflammatory Diseases, Fudan University, Shanghai, 200032 China

**Keywords:** Neurology, Neuroscience

## Abstract

The striatum is one of the first brain regions affected in Parkinson’s disease (PD), where dopaminergic axons projecting from the substantia nigra undergo dying-back degeneration. Growing evidence shows that dopamine depletion triggers network-level remodeling in the striatum, whose pathological significance extends far beyond acute changes in neuronal excitability. Striatal parvalbumin interneurons (PVINs) have recently been recognized as unique integrators of dopaminergic, neuroinflammatory and electrical network signals and as the principal striatal source of glial-cell-line-derived neurotrophic factor (GDNF). This integrative capacity renders PVINs early targets of parkinsonian injury, yet also allows them to orchestrate compensatory plasticity that shapes subsequent disease progression. Here we review how PVINs, via receptor-specific signaling, drive network reorganization in response to dopaminergic degeneration. We propose that these cells follow a compensatory-to-degenerative trajectory that canalizes abnormal synaptic plasticity and thereby exerts a maladaptive influence on PD pathogenesis. Finally, we discuss the therapeutic potential of interventions targeting these adaptive mechanisms.

## Introduction

Parkinson’s disease (PD) is a prevalent neurodegenerative disorder that primarily affects middle-aged and elderly individuals. The main problem of PD is the progressive degeneration of midbrain dopaminergic neurons in the substantia nigra pars compacta (SNc) of the midbrain, which results in motor deficits such as bradykinesia, rigidity, and tremor^1,2^. The striatum (especially the dorsolateral part), which is densely innervated by axonal projections from dopaminergic neurons in the substantia nigra (SN) of the midbrain, is a primary site for early pathological alterations in PD^3^. Evidences indicate that degeneration within the nigrostriatal dopaminergic pathway follows a “dying back” pattern, initiating at striatal synapses and progressing retrogradely along the axons of the medial forebrain bundle (MFB), ultimately affecting the neuronal cell bodies in the SN^4–6^. Thus, the striatum serves as a primary site for the initiation and modulation of PD pathology.

Within the striatum, a rich diversity of neurons forms a complex network. The principal neurons are classically categorized into two major types of spiny projection neurons (SPNs): the direct-pathway SPNs expressing dopamine D1 receptors (D1-SPNs) and the indirect-pathway SPNs expressing dopamine D2 receptors (D2-SPNs)^7^. It is noteworthy that recent transcriptomic and functional studies have revealed a more complex heterogeneity within these populations, suggesting the existence of subtypes that blur this classical dichotomy^8–11^. Among interneurons, those that express the calcium-binding protein parvalbumin (PV), termed PV-positive interneurons (PVINs), are indispensable for finely tuning the activity of both D1-SPNs and D2-SPNs^7,12^. In PD, reduced dopamine levels trigger adaptive alterations within the striatal network. These alterations lead to aberrant circuit activity, including enhanced beta oscillation and synchronized bursting^13,14^.

Strikingly, our recent study in a 6-OHDA-induced PD mouse model identified PVINs as among the earliest victims of parkinsonian pathology. These neurons exhibited somatic shrinkage, downregulated PV protein expression and altered synaptic inputs one week before overt synaptic changes became apparent in SPNs^15^. This early vulnerability could be particularly consequential given the unique roles PVINs play in maintaining striatal homeostasis. For instance, PVINs are the predominant striatal source of glial-cell-line-derived neurotrophic factor (GDNF), a critical mediator of dopaminergic neuron survival, thereby acting as a lifeline for neighboring dopaminergic terminals^16,17^. Simultaneously, they function as non-redundant pacemakers of gamma oscillation^15,18,19^ and master regulators of synaptic excitatory-inhibitory (E-I) balance^20,21^. Consequently, early compromise of PVINs does not merely disrupt a single function; it destabilizes the striatal microcircuitry, unleashing a cascade of network dysfunction that may drive PD progression.

To explore this concept, this review synthesizes recent advances in understanding the functional role of striatal PVINs in PD progression. We begin by summarizing their synaptic inputs, including glutamatergic and GABAergic pathways, and their modulation by neuromodulators (e.g., dopamine, acetylcholine, and serotonin) and neuroinflammatory mediators (e.g., IL-6). We then focus on alterations in PVIN excitability and striatal network dynamics during PD progression. Based on these evidences we propose a novel hypothesis wherein PVINs integrate multiple PD-associated pathogenic cues, disrupt the E-I balance, and thereby drive the pathological evolution of striatal beta oscillations throughout PD development. Finally, we discuss clinically available drugs that recruit PVINs, which could be used to test the hypothesis that such targeting might slow PD progression.

## Striatal PV interneurons: sparse yet indispensable

**Fig. 1:**
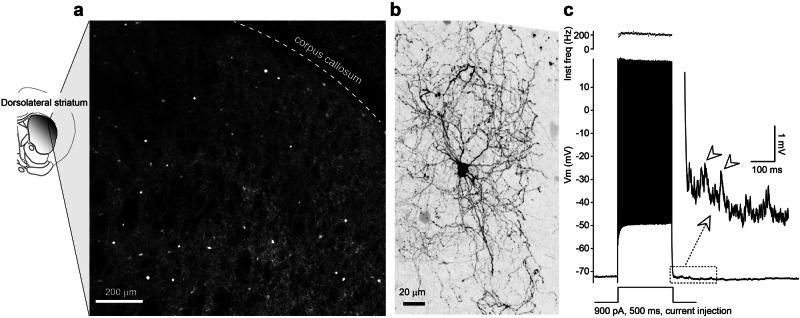
PV interneurons in the striatum. **a** Left, schematic illustrating the ventral-to-dorsal gradient distribution of PV interneurons, showing a higher density in the dorsolateral than in other regions. Right, a confocal image showing PV immunostaining in the dorsal lateral striatum, demonstrating a sparse distribution of somata of PV-positive interneurons. **b** Avidin staining of one striatal PV interneuron displays neurites extending in a basket-like morphology. **c** Representative membrane potential traces from another PV interneuron in response to step-current injection showed action potential firing faster that 200 Hz. Following burst termination, barrages of autaptic events (arrowheads) were immediately observed. The cell was recorded in the presence of 1.5 mM kynurenic acid in the bath to block glutamatergic transmission. Additionally, we used a high-Cl⁻ internal solution, which shifted the reversal potential for Cl⁻ to approximately -15 mV. As a result, the inhibitory autaptic events appeared depolarizing at the resting membrane potential. Under this recording condition, strong current steps evoked high-frequency firing, leading to asynchronous release and allowing autaptic events to be readily identified. The data shown in this figure have not been previously published.

PVINs, comprising around 3-5% of striatal neurons^22,23^, are sparsely distributed throughout the striatum (Fig. 1a). Despite their low density, PVINs deliver the most potent GABAergic inhibition in this region, controlling burst firing, limiting calcium entry, and restricting learning-associated plasticity in SPNs^20,24^. During movement initiation they additionally refine SPN output by selectively silencing task-irrelevant cells, thereby releasing the appropriate ensemble that executes the forthcoming motor sequence^25^. PVINs exhibit distinct electrophysiological traits, including narrow action potentials and high-frequency, non-adapting firing during sustained depolarization, which enable their reliable identification during slice recordings^26^. Their axons form dense basket-like arbors, creating extensive local network that targets nearby SPNs (Fig. 1b). As a result, they are classified as fast-spiking basket cells.

Compared to PVINs in other brain regions, striatal PVINs exhibit several distinctive properties that are particularly noteworthy. For instance, they are capable of synthesizing and releasing GDNF, a unique characteristic absent in nearby cortical or external globus pallidus (GPe) PVINs^27^. Additionally, their presynaptic terminals express the CB1 cannabinoid receptors^28^. This contrasts with the hippocampus, where CB1 receptors are predominantly localized to cholecystokinin-expressing basket cells rather than PVINs^29,30^.

**Fig. 2:**
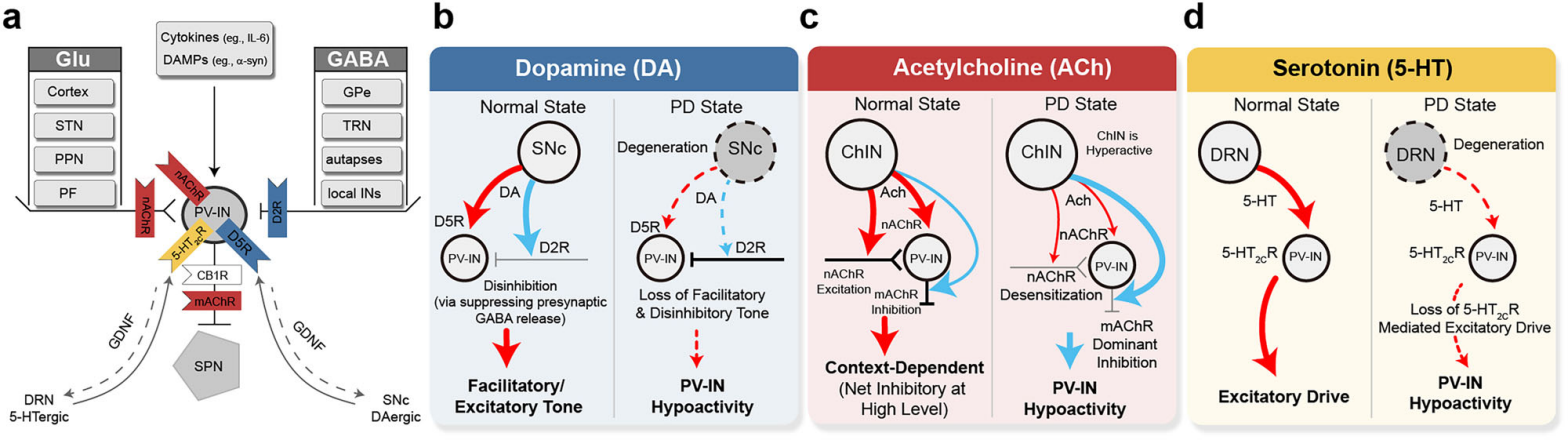
Circuit and modulation of striatal PV interneurons. **a** Schematic illustration of the major synaptic inputs (glutamatergic and GABAergic) and regulatory factors (neuromodulators and neuroinflammatory signals) governing PV interneurons activity and their outputs onto SPNs. Receptors are color-coded, with their signalings pathway detailed in **b**-**d**. **b-d** Summaries comparing the dynamics of key neurotransmitters (dopamine, b; acetylcholine, c; 5-HT, d) under normal and PD conditions, and the resultant convergent drive toward PVIN hypoactivity.

In this section, we summarize four key signaling pathways—synaptic (glutamatergic and GABAergic), neuromodulatory, neurotrophic, and neuroinflammatory—that converge on striatal PVINs, we describe the receptors that mediate each pathway (see Fig. 2). This molecular landscape provides a foundation for understanding PVINs contributions to PD progression.

### Glutamatergic and GABAergic input into striatal PV interneurons

The glutamatergic excitation originates from virtually every region of the forebrain. Cortical regions, particularly the primary somatosensory and motor areas^31^, provide robust monosynaptic excitation through VGlut1+ terminals that uniformly distribute across both proximal and distal dendrites^32^. This cortical input pattern enables a significantly stronger excitatory drive compared to thalamic sources. Thalamic nuclei, including the parafascicular nucleus (PF), contribute VGlut2+ inputs that preferentially target somata and proximal dendrites of PVINs^32^. More recently, the subthalamic nucleus (STN)^33^ and the pedunculopontine nucleus (PPN)^34^ have been identified as additional glutamatergic sources, expanding the subcortical influence on striatal PVINs.

GABAergic inhibition of PVINs arises from both extrastriatal and intrastriatal sources. The GPe^35^ and the thalamic reticular nucleus (TRN)^36^ serve as primary extrastriatal GABAergic inputs. Within the striatum, cholinergic interneurons (ChINs) represent a particularly intriguing source capable of co-releasing glutamate alongside acetylcholine. This co-transmission drives biphasic excitatory postsynaptic currents (EPSCs) in PVINs through a sequential mechanism: an initial direct glutamatergic input from ChINs, followed by presynaptic amplification via nicotinic acetylcholine receptors (nAChRs) located on glutamatergic afferents^37^. The ChIN-mediated circuit extends further through neurogliaform interneurons (NGFs), which receive ChINs innervation and provide slow, persistent inhibition to PVINs. Optogenetic activation of ChINs evokes this slow inhibitory component, which is abolished by GABAA antagonists and prolonged by GABA reuptake blockade, confirming the functional ChIN→NGF → PVIN pathway^37^.

Another often-overlooked synaptic source arises from PVINs themselves—the autapses. Surprisingly, in the sensory cortex, autapses are estimated to contribute approximately 40% of the total inhibitory synaptic conductance in these neurons^38^. This strong self-generated signal serves multiple computational functions: it clamps firing precision^39^, promotes gamma-frequency synchronization^38^, and, during sustained high-frequency bursts, switches to asynchronous GABA release that desynchronizes the local network^40^. This autaptic mechanism has also been identified in striatal PVINs^26^ (see Fig. 1c). Although the reported 40% proportion remains to be confirmed in this region, we show that its role in gamma oscillations resembles that observed in the neocortex^26^. Thus, in striatal circuits, we speculate that this mechanism prevents runaway excitation while maintaining the delicate balance required for precise temporal processing.

The convergence of these excitatory and inhibitory inputs allows PVINs to act as high-fidelity sensors and integrators of overall brain state, translating diverse cortical, thalamic, and intrastriatal signals into precisely timed inhibitory output. Yet the properties of this PVIN-mediated feed-forward inhibition are not dictated by synaptic wiring alone. They are continuously retuned by ambient dopamine, acetylcholine, serotonin and many other neuromodulators. Clarifying how these neuromodulators reset PVIN gain is therefore essential to understand why the same wiring generates healthy gamma rhythm in the intact striatum but pathological beta oscillation after dopamine loss.

### Dopamine, acetylcholine and serotonin signals in striatal PV interneurons

Monoamine neuromodulators, including dopamine, norepinephrine, epinephrine, acetylcholine, serotonin, and histamine, represent a major class of neuromodulators. Among these, the striatum receives especially dense innervation from dopamine, acetylcholine, and serotonin systems^41–43^. Our discussion will therefore primarily concentrate on these three neuromodulators. The roles of the other three—norepinephrine, epinephrine, and histamine—in PD have also garnered increasing attention in recent years. However, due to space constraints, they will not be covered in detail here; interested readers are referred to the specialized literature on these topics^44,45^.

These neuromodulators act on various receptor subtypes located on both the pre- and post-synaptic sites of PVINs. For example, dopamine exerts a two-pronged control: it directly excites PVINs via postsynaptic D5 receptors (D1-like)^46,47^ while simultaneously disinhibiting them via presynaptic D2 receptors that suppress incoming GABAergic terminals^47^. The excitatory effect of D5R, possibly involving cAMP-dependent pathways, may enhance PV protein expression^48^ and contribute to plasticity during motor learning^49^.

Acetylcholine (ACh) in the striatum originates mainly from local ChINs. Viral tracing studies further indicate that extrinsic ACh is supplied exclusively by two brainstem nuclei: the PPN and the laterodorsal tegmental nuclei (LDT)^50^. ACh modulates PVINs through two complementary receptor classes: nicotinic (nAChR) and muscarinic (mAChR) receptors. Both receptor types (α4β2-nAChR^51^ and M1-mAChR^52^) are expressed by PVINs themselves, yet α7-nAChRs^37^ are additionally present on glutamatergic terminals that contact PVINs, where they facilitate glutamate release. In contrast, muscarinic signaling presynaptically inhibits GABA release from PVIN axons^52^. At high ACh concentrations, the net effect may shift toward inhibition as nAChRs desensitize while mAChR-mediated suppression persists.

Serotonin(5-HT), released from raphe nuclei neurons that densely innervate the striatum^41,42^ also regulates PVINs. These interneurons express multiple 5-HT receptor subtypes, most notably 5-HT2C and 5-HT2A. Direct functional evidence shows that activation of 5-HT2C receptor depolarizes PVINs and increases their firing rate^53^. However, little is known about how 5-HT modulates their synaptic activity. Although optogenetic and pharmacological experiments have ruled out a direct role for 5-HT1B receptors in regulating PV-to-SPN transmission^54^, the broader serotonergic modulation remains unclear. Notably, PVINs represent approximately 90% of all striatal neurons expressing 5-HT2A receptors^55^, highlighting their importance as targets for psychoactive drugs that act on this receptor. Despite this, functional studies of 5-HT2A signaling in PVINs are surprisingly sparse. One possible reason is that 5-HT2A receptors are primarily located intracellularly—within organelles such as the Golgi apparatus and endoplasmic reticulum—rather than on the plasma membrane, as recently demonstrated in cortical pyramidal neurons^56^. This intracellular compartmentalization explain why lipophilic psychedelic drugs can readily cross the plasma membrane and activate these receptors to induce behavioral effects, whereas hydrophilic 5-HT cannot. Given that 5-HT is typically at low concentrations within non-serotonergic neurons such as PVINs, it remains an open question whether this implies the existence of endogenous non-5-HT ligands or alternative signaling mechanisms.

Therefore, these findings delineate a balanced modulatory landscape in which dopamine and 5-HT generally promote PVIN activity, whereas high levels of ACh tend to suppress it. In PD, dopamine and 5-HT concentrations fall while ACh levels rise^57,58^. Consequently, these pathological transmitter imbalances converge on a shared phenotype—striatal PVINs hypoactivity—whose detailed characterization will be provided in a later section.

### Neuroinflammatory signal in striatal PV interneurons

Neuroinflammation, a hallmark of PD, represents a double-edged sword: while essential for tissue repair, its dysregulation contributes to neurodegeneration^59,60^. In the MFB 6-OHDA mouse model, rapid dopaminergic denervation is accompanied by glia activation^61,62^ and elevated pro-inflammatory cytokines (e.g., TNF-α, IL-1 and IL-6) in the striatum^62,63^. The similar inflammatory problem have also been observed in MPTP-treated mice^64^, as well as in post-mortem tissue from PD patients^65^. However, it remains unknown whether any established PD animal model is entirely free of similar inflammatory challenges.

Critically, PVINs are vulnerable to this inflammatory milieu, as evidenced by findings in cortical and hippocampal tissue. Their susceptibility is exemplified by the response to IL-6. IL-6 directly induces NADPH oxidase (Nox2) expression and triggers subsequent oxidative stress, leading to decreased expression of PV and GAD67 in cultured cortical PVINs^66,67^. This effect is likely mediated via a non-canonical NFκB pathway: IL-6 binds to its soluble receptor (sIL-6R), forms a complex with the gp130 co-receptor on neurons, and promotes nuclear translocation of NFκB p65 through physical interaction with unphosphorylated STAT3 (U-STAT3), thereby bypassing the classical IκB-dependent mechanism. The resulting U-STAT3/NFκB heterodimer directly enhances the transcription of Nox2 and p22^phox^. Unlike the canonical JAK/STAT3 pathway, this signaling cascade selectively upregulates oxidative stress-related genes without inducing a broad inflammatory response, ultimately leading to superoxide-mediated dysfunction of PVINs^68^.

Beyond cytokine signaling, pattern-recognition receptors, particularly Toll-like receptors (TLRs), constitute a second, parallel inflammatory axis in PD. Based on their subcellular localization, TLRs can be divided into two functional modules: the plasma membrane–associated sentinel module (TLR2/4) and the endosomal sentinel module (TLR3/7/8/9). Both modules are up-regulated in the striatum across multiple PD animal models and cooperate to amplify pathogenic signals^69^. Cell-surface TLR2 is directly activated by extracellular α-synuclein that acts as a damage-associated molecular pattern (DAMP). This activation disrupts autophagy via the AKT/mTOR signaling pathway, leading to the accumulation of α-synuclein in dopaminergic neurons^70^. In contrast, endosomal TLR7/8 are activated by α-synuclein fragments or endogenous microRNAs released during neuronal damage. Their activation promotes dendritic cell maturation, which in turn triggers the infiltration of T cells into the brain and enhances autoimmune attacks against neuronal antigens^71^. Accordingly, genetic deletion of TLR7/8 significantly attenuates the pathological cascade in MPTP-treated mice, from α-synuclein aggregation to dopaminergic neuron loss and ultimately to the manifestation of bradykinesia^71^, underscoring their pathogenic role in PD.

Interestingly, PVINs have recently moved into the TLR spotlight. A recent hippocampal survey showed that ~80% of TLR8-expressing interneurons in CA1 co-express PV, whereas somatostatin- or calretinin-positive interneurons display negligible TLR8 immunoreactivity^72^. TLR8 activation exacerbates neuronal death, offering a plausible explanation for the selective vulnerability of hippocampal PVINs in temporal-lobe epilepsy. Whether this PV-biased expression pattern is conserved in the striatum and whether it sensitizes PVINs to α-synuclein-driven degeneration in PD has yet to be determined.

In summary, PVINs appear to be sensitive to both cytokine- and TLR-mediated inflammatory stress. By translating systemic immune signals into local E-I imbalance, they may act as a cellular bridge between neuroinflammation and the disruption of synaptic plasticity in PD.

### Neurotropic signal by striatal PV interneurons

Striatal PVINs constitute the primary cellular source of GDNF in the striatum, with approximately 95% of GDNF-expressing neurons co-expressing PV^16^. This production is region-specific, as PVINs in the cortex and GPe produce no GDNF^27^. GDNF, discovered in 1993^73^, is a potent protein that promotes the survival and regeneration of dopaminergic neurons within the nigrostriatal pathway. It signals by binding the GFRα1 co-receptor and activates the RET tyrosine kinase^74^, triggering intracellular cascades that enhance neuronal resilience against toxins such as 6-hydroxydopamine (6-OHDA) and 1-methyl-4-phenyl-1,2,3,6-tetrahydropyridine (MPTP)^75^. Pre-clinical studies show that striatal delivery of GDNF protects dopaminergic neurons, elevates dopamine levels, and improves motor performance in rodent and primate models of PD, underscoring its therapeutic potential^76^.

The functional impact of PVIN-derived GDNF is amplified by the spatial arrangement of these interneurons. Electrically coupled and uniformly distributed, PVINs are well-positioned for activity-dependent, widespread release of GDNF throughout the striatum. Moreover, GDNF released by striatal PVINs exerts potent chemoattraction on RET-positive dopaminergic axons over distances up to 7 µm—a range sevenfold greater than that between SPNs and these same axons^77^.

Interestingly, dopaminergic and GDNF signaling pathways converge within PVINs through shared cAMP/PKA cascades. Activation of D1/5 dopamine receptors elevates intracellular cAMP, which triggers PKA-dependent phosphorylation of DARPP-32 and ERK. These molecular events regulate PV plasticity—a process critical for memory consolidation^48^ and implicated in PD progression^15^. The same cAMP/PKA axis also controls GDNF transcription in PVINs, where adenylate cyclase activator forskolin or the membrane-permeable cAMP analog dbcAMP up-regulate GDNF mRNA^27^. Consistently, the GDNF promoter contains canonical cAMP-response elements (CREs), directly linking PKA-mediated CREB phosphorylation to GDNF transcription^27,78^.

Beyond dopamine, GDNF also exerts neurotrophic effects on other neuromodulatory systems. For instance, norepinephrinergic neurons—another class of monoaminergic neurons located in the locus coeruleus—were nearly eliminated 7 months after GDNF ablation in adulthood^17^. In contrast, the cholinergic and GABAergic systems remained unaffected, with no change in cell number observed in these mice^17^. Regarding serotonergic neurons, the role of GDNF has been controversial^73,79^. However, recent research show that 5-HT neurons do express the RET receptor, a key component of the GDNF signaling pathway^80^. Moderate upregulation of endogenous GDNF enhances serotonin release and reuptake, whereas excessive elevation induces an inverted U-shaped response^80^. This dose-dependent effect may reconcile previous conflicting findings. Therefore, these data suggest that striatal PVIN-derived GDNF is a critical modulator that broadly influences the adaptive plasticity of multiple neurotransmitter systems within the striatal circuitry.

Taken together, Striatal PVINs are far more than a fast-spiking brake on SPNs; they occupy the convergence point of neuromodulator, neuroinflammatory, neurotrophic, and electrophysiological signals. Activity-dependent plasticity within these interneurons sculpts striatal assemblies, while their GDNF output preserves the same afferents that govern them, forming a self-reinforcing loop that stabilizes network function in the healthy brain. When PVINs become dysfunctional in PD (as we will see in the next section), this single locus of control unleashes a multi-transmitter avalanche—dopaminergic, noradrenergic, and serotonergic systems all succumb in cascade, underscoring that PD is inherently a multi-transmitter systems disorder.

## Striatal PV interneurons dysfunction during PD progression

Indeed, PVINs are vulnerable to dopamine depletion, as shown by multiple animal models in which striatal PVINs exhibit reduced spiking activity, altered synaptic function, or even cell death, depending on the modeling method. Their hypoactivity has been consistently observed across multiple models. In vivo striatal recordings show that PVIN firing rates decline significantly by 2–3 weeks following 6-OHDA lesioning in mice^15,81^ or in parkin knockout mice^82^. Immunohistochemical analyses support these findings: picrotoxin-induced cortical disinhibition increases the total number of c-Fos+ neurons in the striatum, yet the fraction that are PV + /c-Fos+ decreases, indicating a net suppression of PVINs and, consequently, a loss of feed-forward inhibition that allows SPNs to respond more vigorously to strong cortical drive^83^. Importantly, this hypoactivity is primarily attributed to a shift in synaptic E-I balance—characterized by enhanced inhibitory and more depressed excitatory inputs—rather than intrinsic excitability changes^15^.

Morphologically, PVINs exhibit significant cytological changes, including decreased PV protein expression, transient somatic shrinkage, and rapid, extensive axonal sprouting^15,84^. Interestingly, this sprouting selectively enhances inhibitory control over D2-SPNs, with a doubling in connection probability observed in the MFB 6-OHDA model^84^. Despite their hypoactivity, compensatory axonal sprouting helps maintain overall PVIN output, and GDNF release has also been reported to remain preserved in this model^85^.

However, in some studies, these compensatory mechanisms appear to be overwhelmed, giving way to more severe structural and functional deterioration. In postmortem lenticular nucleus—a subregion of the human striatum—PVIN in PD patients, both LRRK2-mutant and sporadic, showed an ~50% reduction in cell density^86^. In SN 6-OHDA mouse models, viral tracing shows significant loss of PVIN-to-SPN synapses despite preserved PVIN density^87^. In α-synuclein overexpressed rats, striatal PVIN counts decline by 12 months, correlating with worsening motor deficits and reduced GDNF support for dopaminergic neurons^88^. In the MPTP mouse model, degeneration is rapid: PVIN axonal density decreases by day 3 post-treatment, followed by somatic loss at day 7^89^. These observations suggest that these differences may stem from variations in the nature and tempo of pathological insults across models; for instance, the rapid neuroinflammation and oxidative stress induced by acute toxins like MPTP could swiftly overwhelm intrinsic compensatory capacity, whereas the progressive accumulation of protein aggregates (e.g., α-synuclein) in genetic models may gradually disrupt proteostasis and neurotrophic signaling, leading to delayed but sustained neuronal vulnerability.

Across multiple animal models of dopamine depletion and human patients, striatal PVINs exhibit consistent hypoactivity and structural plasticity, but compensatory mechanisms may ultimately fail, leading to synaptic loss and neuronal degeneration.

## A dual-phase model of PV interneurons alteration and striatal network remodeling

**Fig. 3:**
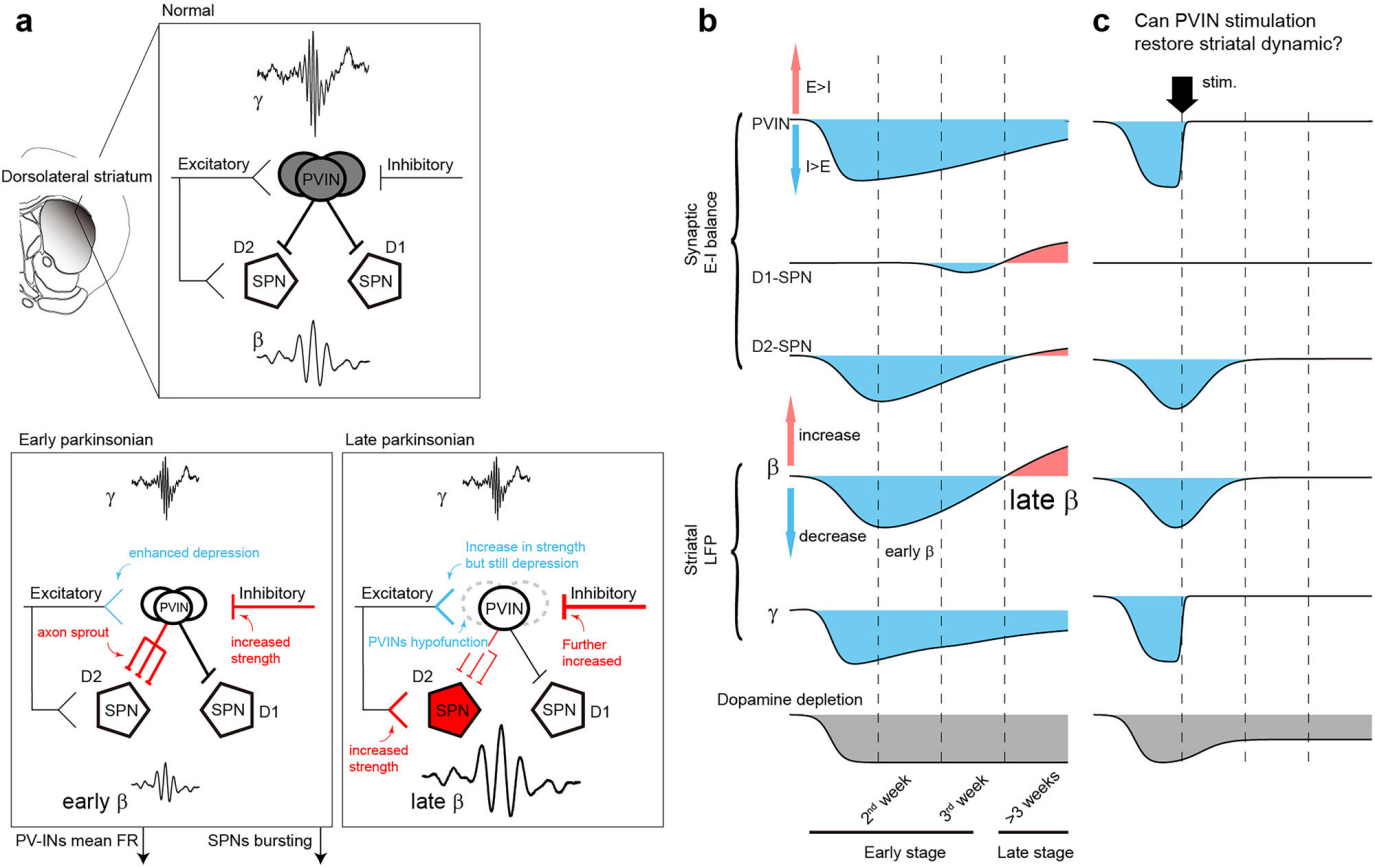
Striatal circuit alterations and the hypothetical role of PV interneurons in PD progression. **a** Schematic summary of longitudinal data from MFB 6-OHDA mouse mode of PD^15^. In the normal intact striatum, both PV interneurons and SPNs receive balanced E-I synaptic inputs, each contributing to the generation of normal gamma or beta oscillations. The direct and indirect pathways mediated by D1- and D2-SPNs are in balance. In the early parkinsonian striatum (Phase I), when dopamine levels reduced, PV interneurons show cytological alternations (reduced soma size, decreased PV expression and axonal sprouting) and receive inhibition-biased synaptic inputs, resulting in diminished gamma oscillation. The sprouting axons of PV interneurons restrict the bursting activity of SPNs, especially D2-positive ones, leading to reduced beta oscillation. Notice that the enhanced inhibition of D2-SPNs counteracts the disinhibition caused by dopamine loss. For D1-SPNs, the dopamine loss‑induced disfacilitation appears insufficient to abolish their intrinsic activity. Therefore, the direct and indirect pathways remain balanced at this stage. Thus, PV interneuron‑driven network changes mask the early pathology caused by dopamine depletion, which may correspond to the latent period before motor symptoms emerge. At later stages (Phase II), PV interneurons become hypofunctional (loss in cells or synaptic innervations onto SPNs) and release SPNs from inhibition, resulting in enhanced beta oscillations. The dopamine system (along with other modulator systems, see main text) undergoes accelerated degeneration due to loss of trophic support from GDNF normally released by PV interneurons. At this point, the direct and indirect pathways finally become completely unbalanced. Blue color indicates a functional decrease and red color indicates an increase. **b** Schematic curves showing the time course of synaptic E-I balance and network activity alternations following 6-OHDA injection in MFB. **c** Hypothesis: We hypothesize that stimulating PVINs within an early time window might suppress their own aberrant axon sprouting, subsequently normalize pathological synaptic changes by rebalancing the E-I ratio, and thereby restore LFP activity across the striatal network. This strategy might also increase dopamine levels in the striatum by promoting the regeneration of dopaminergic axons via enhanced GDNF production.

Based on these observations, we propose a dual-phase model of PVIN alterations that drive striatal remodeling during PD progression (see Fig. 3). This model posits that PVINs undergo two distinct phases—an initial compensatory phase followed by a degenerative phase. Each phase differentially orchestrates the disease progression of network dysfunction by shaping the maladaptive trajectory of E-I synaptic plasticity. It should be noted, however, that this model is primarily derived from the MFB 6-OHDA lesion paradigm, which, to our knowledge, is the only one with longitudinal data on PVINs throughout PD progression. Consequently, the proposed dual-phase scheme must be refined with future longitudinal data from other animal models or PD patients.

Phase I (Early/Compensatory Phase): This phase is characterized by PVIN hypoactivity alongside structural plasticity. Despite reduced firing rates and somatic shrinkage, PVINs exhibit extensive axonal sprouting that sustains their overall inhibitory output. Notably, this sprouting preferentially targets D2-SPNs of the indirect pathway, which are crucial for suppressing competing motor programs. Functionally, this suppresses burst firing in SPNs and transiently attenuates beta oscillations in the striatum, while gamma oscillations are diminished as a result of general PVIN hypoactivity. These changes are adaptive in nature, potentially serving to stabilize striatal output in the face of dopaminergic loss. Behaviorally, this early compensatory phase might correspond to a preclinical or prodromal stage, in which fine motor adjustments or cognitive strategies mask underlying network instability before the emergence of overt motor signs. The preferential inhibition of D2-SPNs by PVIN helps maintain the balance between direct and indirect pathways temporarily, thereby preventing an early bias towards the hyperdirect pathway dominance that favors beta-oscillation generation within the cortico-basal ganglia network.

Phase II (Late/Degenerative Phase): In the second phase, PVINs either undergo apoptosis or lose synaptic efficacy, depending on the PD model. This may result from the sustained oxidative stress associated with neuroinflammatory signaling detailed in previous section, ultimate overwhelming compensatory mechanisms. The attenuation or loss of PVIN-mediated inhibition further shifts the E-I balance in SPNs toward excitation, amplifying the changes initiated in Phase I. Consequently, SPN excitability increases, particularly in D2-SPNs, which were the primary targets of sprouting PVIN axons in the earlier phase. The disinhibition of D2-SPNs leads to excessive activity of the indirect pathway, resulting in increased inhibition of the thalamus and a consequent reduction in motor output, contributing to akinesia and bradykinesia. D1-SPNs may also exhibit increased excitability due to the general loss of PVIN-mediated inhibition, albeit to a lesser extent than D2-SPNs in this model framework; however, the loss of dopaminergic D1 receptor stimulation may limit their ability to fully engage the pro-kinetic direct pathway. Thus, the net effect is a dominant hyperactivation of the indirect pathway alongside a dysfunctional direct pathway, creating a pathological imbalance that underlies core motor deficits. At the network level, this disinhibition leads to exaggerated beta oscillation, a hallmark of advanced PD.

Importantly, the specific manifestations of these phases depend on lesion severity and model type. For example, direct intrastriatal 6-OHDA produces greater local damage and triggers both astrogliosis and microgliosis, whereas intra-MFB delivery limits striatal injury to astrogliosis alone^61^. Paradoxically, the milder MFB lesion transiently suppresses striatal beta- and gamma-band oscillations—an effect absent after the more destructive striatal injection^90^. This highlights that oscillatory phenotypes are not linearly linked to visible tissue damage, much like how computational models require parameter tuning to reproduce specific network activities. Moreover, classical 6-OHDA lesions generally do not induce striatal PVIN loss, although exceptions exist^91^. In contrast, more aggressive models—such as MPTP exposure^89^ or α-synuclein overexpression^88^—show PVIN degeneration more frequently, though findings remain inconsistent across studies^16^. While some of these patterns may reflect aspects of advanced PD pathology^92^, the extent of PVIN vulnerability appears to vary with the model and experimental conditions. Thus, the proposed dual-phase model reflects a severity-dependent continuum in which early functional impairment can evolve into structural degeneration, accompanied by corresponding shifts in network-level biomarkers such as local field potential (LFP) oscillations.

### Strengths and weaknesses of the dual-phase model

The classical “dying-back” hypothesis proposes that PD pathogenesis begins at the dopaminergic axon terminals in the striatum. Given that PVINs are highly sensitive to dopamine loss, their deficient neurotrophic support—particularly reduced GDNF release—may drive this retrograde progression. This reduction in GDNF subsequently further impairs dopamine as well as other GDNF-dependent modulator systems, including norepinephrine and serotonin pathways, thereby exacerbating and amplifying the overall degenerative process. Building on this perspective, we proposed that PVIN in the striatum should be a key player during the disease progression of PD. The dual-phase model offers a conceptually simple and mechanistically grounded framework for understanding how these neurons contribute to PD pathogenesis. By integrating neurochemical, neuroimmune, neurotrophic, and electrophysiological changes into a self-organized process, the model provides a coherent explanation for the complex evolution of PD pathology. Moreover, grounding the model in synaptic plasticity might allow it to be naturally extended to explain motor-learning deficits, a core feature of PD that stems from aberrant synaptic plasticity^93,94^.

One particularly important implication of the model is its potential to explain the well-documented gender disparity in PD incidence. While PD affects both sexes, men exhibit significantly higher incidence rates and earlier onset than women^95^. This disparity may be partly attributed to the neuroprotective effects of estrogen. In 6-OHDA-lesioned animal models, estrogen has been shown to confer substantial protection against dopaminergic neuron loss, particularly in cases of partial lesions^96^. Notably, estrogen also upregulates PV expression^97^. Consistent with this, sexual dimorphism in PVIN density has been observed across multiple brain regions^98,99^, including the striatum^100,101^, where female rats exhibit significantly higher numbers of PVINs than males^100^. These findings strongly suggest that estrogen-mediated upregulation of PV contribute to neuroprotection during the early stages of PD.

Despite these strengths, the model also faces notable limitations. Most critically, its empirical foundation is largely derived from longitudinal studies in the MFB 6-OHDA mouse model, with limited cross-validation in other PD models. Additionally, the phenotypic signatures distinguishing Phase I from Phase II are often subtle and highly dependent on lesion severity and type. For example, the model predicts a *decrease*—rather than an increase—in striatal beta oscillations during early PD stages. This prediction is challenging to validate in human patients, especially since striatal field potentials are seldom recorded prior to motor symptom onset. Future directions to address these gaps should focus on two fronts: First, developing the model’s mathematical framework to help identifying more robust biomarkers, and improving assays for longitudinal tracking of synaptic E‑I balance in other animal models. Second, incorporating PVIN‑specific PET tracers in future trials will be crucial for translational validation and enhancing the model’s predictive utility.

### Connection with other frameworks of PD progression

This dual-phase model proposed here is certainly not an exclusive theory of PD progression. Instead, it offers a complementary perspective that intriguingly intersects with several established models, as explored in the following discussion.

First, when considered alongside the classic neurocentric, dopamine-first model that attribute PD progression to the thresholds of dopamine loss^1^, the PVIN-centric view provides a plausible substrate for the non-linear relationship between dopaminergic depletion and clinical symptoms. As detailed in Fig. 3, once dopamine levels drop, the expected disinhibition of D2-SPNs is offset by a parallel sprouting of PVIN axons that clamps down on their burst firing. This compensatory masking mechanism likely explains the absence of overt symptoms during the prodromal phase. Furthermore, the system’s remarkable compensatory capacity, where behavioral phenotypes emerge only after substantial dopamine loss^102^, may be shaped by the PVIN-mediated, GDNF-dependent integration of multiple transmitters, which dictates how cortico-basal ganglia network respond to diminishing striatal dopamine. This circuit-level perspective helps explain why symptom severity does not always linearly correlate with dopamine depletion and how network-level dysfunction, such as pathological oscillations, emerges.

Second, when juxtaposed with the α-synuclein-centric model that depicts PD progression as the topographic, prion-like spread of α-synuclein aggregates^103^, the PVIN model acts as a downstream circuit mechanism through which diffuse proteinopathy translates into specific network dysfunction. PVIN across various brain regions appear particularly vulnerable to α-synuclein. In SNCA transgenic rats (which overexpress α-synuclein), there is a progressive loss of PVIN (but not ChINs), along with a dramatic reduction in GDNF expression in the striatum^88^. In human patient at Braak stages 3–5, PVIN (but not somatostatin-positive interneurons) in the amygdala show higher colocalization with α-synuclein aggregates and a significant reduction in density^104^. Moreover, α-synuclein overexpression in the motor cortex induces ferroptosis (an iron-dependent form of cell death) specifically in cortical PVINs^105^. These findings suggest a strong involvement of PVIN in α-synucleinopathy. Of note, since striatal PVINs receive direct glutamatergic projections from the PPN (a region affected early in Braak stage 3 that significantly impacts rapid eye movement (REM) sleep in patients), PVINs may be among the first circuit elements to detect and be influenced by pathology originating in the PPN. It is unknown whether PPN-derived signals modulate the PVIN-mediated remodeling process during sleep.

## Non-dopaminergic chemical strategies that recruit PV interneurons

According to our hypothesis, the maladaptive hypoactivity of striatal PVINs and the associated collapse of gamma oscillation could be viewed as an early causal node in PD. If this node can be reversed during the prodromal or early-clinical window, the entire cortico-basal ganglia network might be redirected away from irreversible degeneration. Here we highlight two drug classes that already possess human safety data and whose beneficial effects in PD models coincide with direct excitation of PVINs: (i) nicotine and (ii) AMPA-receptor positive allosteric modulators (AMPAkines).

Nicotine has long been recognized as a neuroprotective agent, but its therapeutic window appears narrow and confined to early stages. In both 6-OHDA rat and MPTP monkey models, prophylactic nicotine administration mitigated motor impairment and neuronal loss, whereas treatment initiated after substantial dopaminergic degeneration was ineffective^106^. Similarly, in humans, nicotine exposure (e.g. cigarette smoking) is associated with a reduced PD risk in individuals under 75 years of age, but this protection disappears in those over 75^107^. These data indicate that nicotine’s neuroprotection is restricted to early disease stages and is insufficient to counteract age-related risk or advanced pathology. The molecular targets for nicotine remain unclear because its primary striatal subunits, α4β2 and α7, contribute little^108^. The cellular target, however, become clear. Electrophysiological studies confirm that 100% of striatal PVINs exhibit nicotinic currents upon exposure^109^. This is in stark contrast to other striatal neuron types, such as SPNs (~32%), ChINs (~44%), and low-threshold spiking interneurons (~33%)^109^. Thus, striatal PVINs represent a non-redundant cellular substrate for nicotine action. Nicotine therefore serves as a first-in-class pharmacological tool for testing whether recruitment of PVINs during the compensatory Phase I can alter disease trajectory and delay the transition into Phase II.

AMPAkines (e.g., CX516, LY503430, LY404187 and many others) represent a novel strategy for modifying PD progression by leveraging intrinsic neurotrophic pathways (BDNF/GAP-43)^110^ and rebalancing cortical-striatal circuitry^111^. Unlike dopaminergic agents, AMPAkines do not acutely alter baseline or L-DOPA-induced rotational behavior^110^, indicating their role as disease-modifying agents rather than symptomatic treatments. Interestingly, in 6-OHDA-lesioned rats, even delayed AMPAkine treatment initiated after nigral dopaminergic cell death still promoted functional recovery^112,113^, supporting true disease-modifying effects beyond mere neuroprotection. Recent work in epilepsy models shows that CX516 exerts its anti-seizure effect primarily through striatal PVINs^114^. Both intraperitoneal and intrastriatal delivery markedly suppress spike-wave discharges (SWDs)^114^—a LFP pattern resembling the high-voltage spindles (HVS) described in PD^115–117^. Although AMPARs are widely expressed, PVINs exhibit the highest firing rates in the striatum; thus, their high gain in response to AMPAkines is likely to dominate the overall therapeutic outcome. We therefore propose that AMPAkines remodel corticostriatal circuits largely through activation of PVINs, thereby slowing PD progression particularly if administered before extensive degenerative Phase II changes occur. Notably, AMPAkines compounds, including CX516 and CX691, have accumulated substantial clinical data from trials across diverse neuropsychiatric disorders such as schizophrenia and Alzheimer’s disease^118^. Building upon this foundation, the repurposing of AMPAkines for PD treatment offers a strategically feasible and accelerated path for translational testing.

In summary, epidemiological, electrophysiological, and translational evidence positions both nicotine and AMPAkines as promising, low-risk candidates to test whether PVIN recruitment can halt or slow PD progression.

## Conclusions

In conclusion, a growing body of evidence suggests that striatal PVINs act as early sentinels of dopaminergic stress. Their unique molecular toolkit allows them to integrate dopaminergic, immune and trophic cues and to shape E–I balance. Whether they also act as active drivers and propagators of parkinsonian remodeling process remains an open question. Future work must determine whether these interneurons actively trigger the non-cell-autonomous synaptic maladaptation underlying parkinsonian circuit dysfunction, rather than such dysfunction arising merely from cell-autonomous responses to dopamine loss. Establishing this causality would make nicotine or AMPAkine-mediated recruitment of PVINs a ready-to-test strategy to reset network dynamics before degeneration, thereby shifting therapeutic focus from dopamine replacement to circuit preservation.

## Data Availability

The datasets used and/or analyzed during the current study are available from Q.H. or X.W. on reasonable request.
